# Response gene to complement 32 promotes tumorigenesis by mediating DNA damage repair and inhibits CD8+ T cells infiltration in diffuse large B-cell lymphoma

**DOI:** 10.3389/fimmu.2025.1591615

**Published:** 2025-07-16

**Authors:** Xiyuan Zhang, Tiange Lu, Chunlei Shi, Mengfei Ding, Ling Wang, Xinting Hu, Xin Wang

**Affiliations:** ^1^ Department of Hematology, Shandong Provincial Hospital, Shandong University, Jinan, Shandong, China; ^2^ Department of Hematology, Qingdao Central Hospital, University of Health and Rehabilitation Sciences, Qingdao, China; ^3^ Department of Hematology, Qingdao Traditional Chinese Medicine Hospital, Qingdao Hiser Hospital Affiliated of Qingdao University, Qingdao, China; ^4^ Department of Hematology, Shandong Provincial Hospital Affiliated to Shandong First Medical University, Jinan, Shandong, China

**Keywords:** response gene to complement 32 (RGC32), diffuse large B cell lymphoma (DLBCL), cell cycle, DNA damage repair (DDR), CD8+ T cells

## Abstract

**Background:**

Response gene to complement 32 (RGC32), a complement activation-inducible factor broadly expressed in normal human tissues, has been implicated in tumorigenesis through its dysregulated expression in various malignancies and its involvement in critical oncogenic processes. Despite its established roles in cancer biology, RGC32 remains uncharacterized in diffuse large B-cell lymphoma (DLBCL). This study provides the first comprehensive investigation of RGC32 expression patterns and functional contributions to DLBCL pathogenesis, elucidating its potential as a novel therapeutic target or prognostic biomarker in this disease.

**Methods:**

Immunohistochemical (IHC) staining of RGC32 was performed on specimens from 32 Reactive hyperplasia lymphoid (RHL) patients and 80 DLBCL patients. To evaluate the role of RGC32 in DLBCL, lentivirus vectors either encoding shRGC32 or shControl were transfected into DLBCL cell lines. RNA-sequencing (RNA-seq) analysis was performed between shRGC32 and shControl stably transfected OCI-LY1 cells and functional enrichment analyses used gene ontology (GO) and kyoto encyclopedia of genes and genomes (KEGG). In order to explored its functions *in vivo*, xenograft models were established by subcutaneously injecting shRGC32 and shControl transfected DLBCL cells into SCID beige mice.

**Results:**

Immunohistochemical analysis revealed RGC32 overexpression in DLBCL tissues contrast with RHL, and was associated advanced Ann Arbor stage (p = 0.043), B symptoms (p = 0.020), and poor progression-free survival (p = 0.015) and overall survival (p = 0.035). Functional studies demonstrated that RGC32 knockdown via shRNA significantly suppressed DLBCL cell proliferation *in vitro* and *in vivo*, with xenograft models showing reduced tumor growth and Ki-67 expression. RNA-seq analysis linked RGC32 depletion to downregulation of cell proliferation and impaired DNA damage repair (DDR) mechanisms. Western blot showed RGC32 knockdown could suppress ATM/ATR/CHK1 pathway and increase the tumor mutational burden (TMB). Furthermore, after inhibition of RGC32, infiltration of CD8+ T cells was increased in DLBCL tumor microenvironment (TME).

**Conclusions:**

This study highlights that RGC32 is a novel molecule in DLBCL progression and might be a potential therapeutic target for DLBCL therapy.

## 1 Introduction

As the predominant form of non-Hodgkin lymphoma (NHL) observed in adult populations, diffuse large B-cell lymphoma (DLBCL) is characterized by its rapid progression and high malignant potential (1). Although the application of monoclonal antibodies and antibody-drug conjugates, the prognosis of relapsed and refractory DLBCL patients are still poor (2). DLBCL is a highly heterogeneous disease, therefore, it is needed to figure out more available biomarkers for DLBCL treating.

Response gene to complement 32 (RGC32) also known as the complement 32 response gene and C13orf15, is an important complement response gene (3). RGC32 has no homology with other human proteins, but it has been demonstrated to be a binding partner and substrate of p34CDC2, and is related to centrosome formation during mitosis (4). Therefore, RGC32 can regulate the cell cycle and promote cell proliferation. Aberrant expressions of RGC32 can be found in several human cancers and a growing number of data have confirmed the significance of RGC32 in the cancer development (3). Furthermore, RGC32 has been identified as a critical modulator of malignant cell proliferation (5), epigenetic modifications (6) and metastasis (7) in different tumors, and it is a promising therapeutic target in the future. However, the role of RGC32 in DLBCL is still unknown. We hypothesized that RGC32 is likely involved in the biological process of DLBCL.

DNA damage repair (DDR) is a series of cellular responses to DNA damages, including DNA damages removing, DNA damage checkpoints activating, cell cycle arresting and apoptosis inducing (8). Recent studies have found that DDR is closely related to immune infiltrations in tumor microenvironment (TME) (9). Dysfunction of DDR enhances antigen processing and presentation via elevated tumor mutational burden (TMB) and neoantigen accumulation (10). At the same time, DDR also can influence the immune escape by regulating the ligand of programmed death 1 (PD-1), which identified as PD-L1 (11). Furthermore, patients with DDR injury are sensitive to immunotherapy (9) and have a much better prognosis (12).

Our present research was focused on evaluating the clinical relevance and functional implications of RGC32 in DLBCL. RGC32 expression is found to be upregulated in DLBCL and is associated with unfavorable prognosis. Inhibition of RGC32 displayed anti-lymphoma effects through suppression of cell cycle and DDR pathways, and also could promote CD8+ T cells infiltration in TME of DLBCL. These findings imply that RGC32 may serve as a novel promising molecule in DLBCL progression and a potential therapeutic target for DLBCL treatment.

## 2 Materials and methods

### 2.1 Patient samples and cell lines

This retrospective cohort study analyzed formalin-fixed, paraffin-embedded (FFPE) tumor specimens from 80 DLBCL patients treated with R-CHOP regimen between 2011-2022. The detailed information of the DLBCL patients could be found in Additional file 1. Another 32 reactive hyperplasia patients’ tissues serving for control were obtained at the same time. Histological diagnoses were established in accordance with the 5th classification of the World Health Organization (WHO) (13). Peripheral blood mononuclear cells (PBMCs) were extracted from peripheral blood samples donated by 8 healthy volunteers. The experiment was complied with the Declaration of Helsinki and all samples were obtained with the participants’ informed consent. The Medical Ethics Committee of Shandong Provincial Hospital reviewed and approved all the study plans. Human DLBCL cell lines including OCI-LY1, OCI-LY8, OCI-LY10, U2932 and VAL were purchased from ATCC and maintained in IMDM medium (Gibco, CA, USA) containing 10% fetal bovine serum (Gibco, CA, USA) under standard culture conditions (37°C, 5% CO2 humidified atmosphere).

### 2.2 Hematoxylin-eosin staining and immunohistochemical

Formalin-fixed paraffin-embedded tissue sections were generated following established protocols for H&E staining and IHC analysis, as detailed in Additional file 2. Histopathological evaluation of DLBCL specimens employed a two-parameter scoring system: staining intensity grading (0 = negative; 1 = weak; 2 = moderate; 3 = strong) multiplied by positive area proportion scoring (0 = <5%; 1 = 5-25%; 2 = 25-50%; 3 = 50-75%; 4 = ≥75%). Specimens were stratified into low-expression (0-7) and high-expression (8-12) groups based on composite score thresholds. The antibody panel comprised anti-RGC32 antibody (Biorbyt, orb2372, 1:200), human anti-CD8a antibody (ABclonal, A0663, 1:200), mouse anti-CD8a antibody (Servicebio, GB114196, 1:500), and anti-Ki67 antibody (Servicebio, GB121141, 1:300).

### 2.3 Western blot

Western blot analysis of DLBCL cell lysates was performed according to standardized methods described in Additional file 2 (14). Primary antibodies employed in this investigation comprised RGC32 (NOVUS, NBP2-93098, 1:1000), GAPDH (Zhongshan Goldenbridge, TA-08, 1:2000). Additional markers including c-myc (18583), Cyclin D1 (2922), CDK4 (12790), p27 (3688), p-ATM (5883), p-ATR (2853), p-CHK1 (2348), IRF1(8478), PD-L1(13684), p-H2AX (9718) were all purchased from Cell Signaling Technology (Beverly, USA), and the dilution used for all antibody was 1:1000.

### 2.4 Poly(A) tail length assay

This assay was carried out by Poly(A) Tail-Length Assay Kit (76455, Affymetrix, USA) according to the instruction. The specific PCR forward primer of RGC32 was referred to previously published literature (15). Gene-specific amplification was performed using a forward primer targeting the RGC32 sequence paired with a reverse primer complementary to the region immediately upstream of the Poly(A) initiation site, enabling precise Poly(A) tail length quantification (primer details in Additional file 2). Amplified products were resolved by 2% agarose gel electrophoresis, with tail lengths quantified through comparative gel analysis.

### 2.5 Cell transfection

The OCI-LY1 and OCI-LY10 cell lines underwent genetic modification through transfection with RGC32-specific siRNA and corresponding control plasmids (provided by GeneChem, Shanghai). This procedure was conducted following standardized protocols at an optimized multiplicity of infection (MOI) of 50. Subsequent to the 72-hour transfection period, stable transformants were isolated through antibiotic selection using 2 μg/ml puromycin. Transfection efficacy verification was performed through protein expression analysis of RGC32 via Western blotting.

### 2.6 Cell proliferation and cell cycle assay

*In vitro* cell proliferation assessment was conducted utilizing the CCK-8 detection system (Yeasen Biotechnology). Lentivirus-transfected DLBCL cells were cultured in 96-well plates and subjected to kinetic monitoring through CCK-8 reagent addition (10 μl/well) at predetermined intervals (0, 24, 48, 72, 96 hours). Following a 4-hour incubation at 37°C, spectrophotometric measurements were performed at 450 nm using a Multiskan GO microplate analyzer (Thermo Scientific, USA). Parallel cell cycle profiling was implemented through propidium iodide staining methodology, with quantitative analysis executed on a Navios flow cytometer (Beckman Coulter, CA, USA) following established protocols.

### 2.7 Xenograft tumor models *in vivo*

The experimental protocol was approved by the Animal Care and Research Advisory Committee at Shandong Provincial Hospital, with all procedures conducted in accordance with institutional guidelines. Female beige SCID mice (age: 4 weeks; source: Weitong Lihua Laboratory Animal Center, Beijing) were randomly divided through simple randomization into two experimental groups. These groups received subcutaneous injections of either shRGC32-transfected or control vector-transfected DLBCL cells (1×10^7^ cells/mouse) into the right hind limb to establish xenograft tumor models. Tumor progression was monitored biweekly using digital caliper measurements for volumetric assessment. Following a 3–4 weeks experimental period, all animals underwent subsequent histopathological evaluation of tumor specimens.

### 2.8 RNA-sequencing

Total RNA was isolated using RNAiso Plus reagent (Takara, Dalian, China) from three independent replicates of OCI-LY1 cells stably transduced with either shRGC32 or shControl constructs. Following library preparation through reverse transcription, comparative transcriptome profiling was performed using DESeq2 (v1.20.0) in R environment to identify statistically significant differentially expressed genes between experimental groups. To elucidate the biological implications of these transcriptional changes, functional enrichment analyses were subsequently conducted, encompassing Gene Ontology (GO) categorization and Kyoto Encyclopedia of Genes and Genomes (KEGG) pathway annotation.

### 2.9 Tumor-infiltrating lymphocytes analysis

Xenograft tumors established by subcutaneously injected OCI-LY1 cells with shRGC32 vectors or empty control vectors stable transfected. Details of steps were provided in Additional file 2. Tumor pieces were digested by collagenase IV (Solarbio, China) and separated by Percoll gradient (Solarbio, China). Cell surface CD8a and CD3 of TILs was stained with CD8a Flow antibody (Proteintech, PE-65069, 0.3μl) and CD3 Flow antibody (Proteintech, APC-65077, 0.5μl), and tested by FACS- 240 Navios Flow Cytometer (Beckman Coulter Inc. USA).

### 2.10 Quantitative real-time PCR

Total RNA extraction was performed with RNAiso Plus reagent (Takara, Dalian, China). RNA quantification was conducted using a Nanodrop 2000 spectrophotometer (Thermo Fisher Scientific, Waltham, MA, USA). Complementary DNA synthesis was achieved through reverse transcription with commercial kits (Vazyme, Nanjing, China). Subsequent quantitative PCR analysis was performed on a Light Cycler 480II platform (Roche, Basel, Switzerland) employing SYBR Green Premix Ex Taq II reagents (Vazyme, Nanjing, China). All primer sequences for target amplification were documented in Additional file 2, with GAPDH serving as the endogenous reference gene for normalization. Relative gene expression levels were determined through comparative threshold cycle analysis using the 2^−ΔΔCt^ calculation method.

### 2.11 Statistical analysis

Statistical analyses were conducted using SPSS 24.0 (IBM Corporation, USA) and GraphPad Prism 5.0 software. Data obtained from *in vitro* experiments are expressed as mean ± standard deviation (SD) from three independent replicates. Survival curves were constructed using Kaplan-Meier analysis, and intergroup differences were assessed with the log-rank test. Statistical comparisons were performed using Student’s t-test and one-way analysis of variance (ANOVA). A p-value threshold of 0.05 was established for statistical significance, with asterisks denoting specific probability levels (*p < 0.05, **p < 0.01, ***p < 0.001).

## 3 Results

### 3.1 Elevated RGC32 expression in DLBCL correlated with adverse clinical outcomes

**Figure 1:**
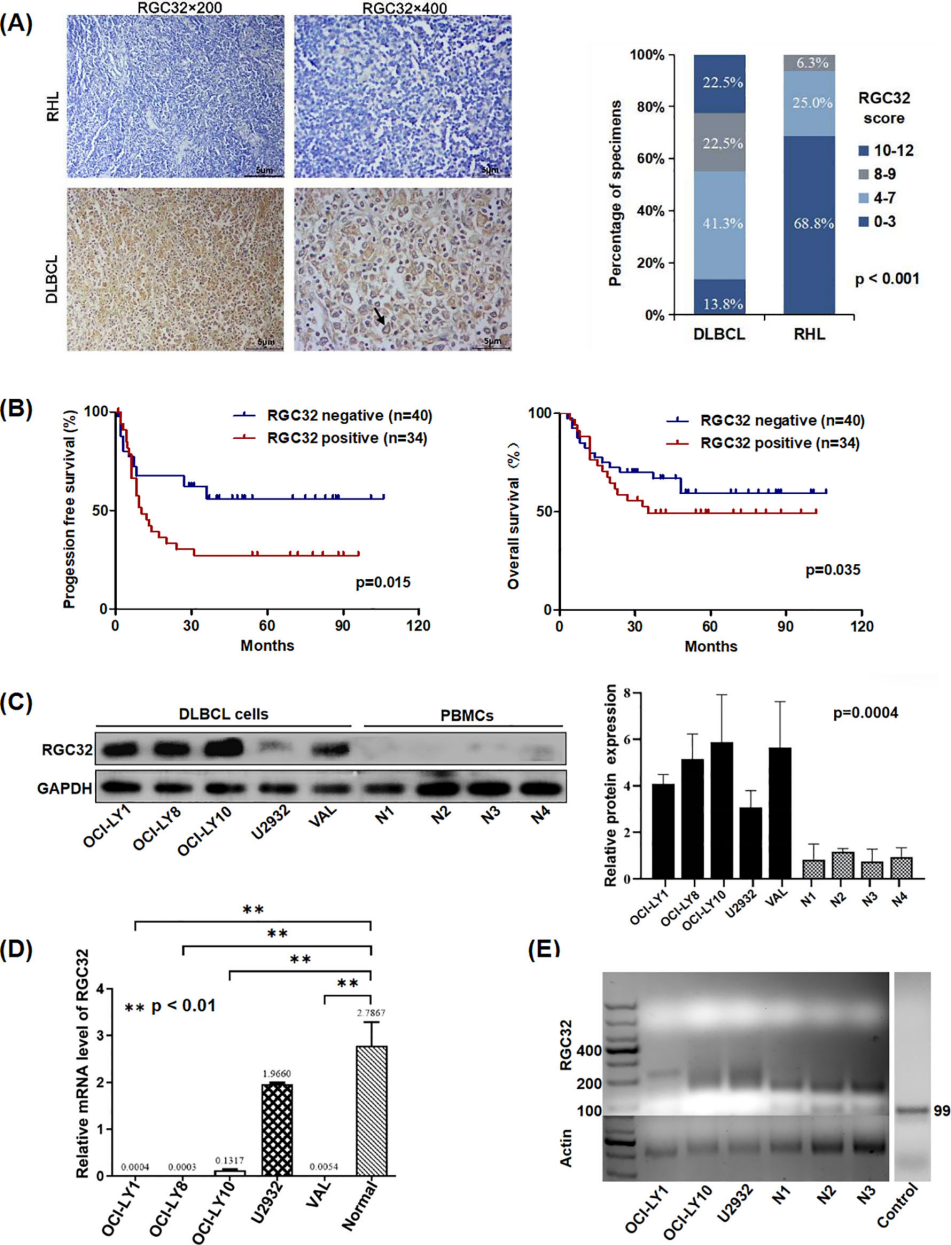
RGC32 expression was up-regulated in DLBCL and was related with adverse progression. **(A)** Immunohistochemical staining of RGC32 in DLBCL (n = 80) and RHL (n = 32) tissues. Bar = 5μm. **(B)** RGC32 expression in DLBCL was correlated with progression free survival and overall survival based on Kaplan-Meier survival curve analysis of IHC datas. **(C)** Protein expressions of RGC32 in DLBCL cell lines and PBMCs was detected by Western blotting. **(D)** The expression of RGC32 mRNA in PBMCs and DLBCL cell lines by RT-PCR. **(E)** The PAT length of RGC32 mRNA in PBMCs and DLBCL cell lines.

To elucidate the functional role of RGC32 in DLBCL pathogenesis and clinical outcomes, IHC analysis was conducted to compare RGC32 expression patterns between DLBCL specimens and RHL controls. Cytoplasmic localization of RGC32 was predominantly observed in DLBCL tissues. Quantitative evaluation revealed markedly elevated RGC32 protein levels in DLBCL compared to benign lymphoid hyperplasia (**Figure 1A**), implicating its potential association with disease progression in this lymphoma subtype. RGC32 positivity was detected in 45% (36/80) of DLBCL cases, contrasting with only 6.3% (2/32) in RHL controls (p < 0.001).

Clinical characteristics correlation analysis (**Table 1**) demonstrated significant associations between RGC32 expression and advanced Ann Arbor stage (p = 0.043) as well as B symptoms (p = 0.020). While a trend toward increased RGC32 positivity was observed in patients with extra-nodal involvement, this did not reach statistical significance (p = 0.052). Survival analysis via Kaplan-Meier curves revealed that DLBCL patients with elevated RGC32 expression exhibited significantly reduced progression-free survival (PFS; p = 0.015) and overall survival (OS; p = 0.035) compared to RGC32-negative counterparts (**Figure 1B**). Additionally, comparative analysis revealed significantly elevated RGC32 protein expression levels in DLBCL-derived cell lines when compared with PBMCs isolated from healthy control subjects (**Figure 1C**). Collectively, these data suggest that RGC32 overexpression may serve as a novel prognostic biomarker for monitoring disease progression in DLBCL patients.

**Table 1:** Clinical characteristics of the RGC32 expression in DLBCL patients.

Characteristics	Patients (N)	negative (N)	positive (N)	P value
Age(years)				
<60	29	15	14	0.644
≥60	51	29	22	
Gender				
Male	45	23	22	0.434
Female	35	21	14	
Ann Arbor Stage				
I/II	28	19	9	**0.043**
III/IV	48	21	27	
IPI score				
0-2	40	22	18	0.668
3-5	36	18	18	
Subtype				
GCB	32	20	12	0.402
Non-GCB	40	21	19	
B symptom				
No	54	33	21	**0.020**
Yes	22	7	15	
Elevated LDH				
Yes	36	18	18	0.668
No	40	22	18	
Extranodal involvement				
Yes	53	24	29	0.052
No	23	16	7	

The bold values represents statistically significant results (p<0.05). IPI, international prognostic index; GCB, germinal center B-cell; Non-GCB, non-germinal center B-cell; LDH, Lactate dehydrogenase.

### 3.2 RGC32 mRNA in DLBCL had longer Poly (A) tails and higher transcription efficiency compared with PBMCs cells

Interestingly, while RGC32 protein expression was lower in PBMCs of healthy donors compared to DLBCL cell lines (**Figure 1C**), its mRNA levels showed an inverse pattern with significantly higher expression in healthy PBMCs (**Figure 1D**). This mRNA-protein discordance aligns with prior reports on RGC32’s post-transcriptional regulation, which has been attributed to variations in Poly(A) tail length (15). Given the critical role of Poly(A) tails in maintaining mRNA stability and modulating translational efficiency (16), we verified this phenomenon in DLBCL using a Poly(A) tail length assay. Our analysis revealed distinct electrophoretic patterns: discrete bands corresponding to shorter Poly(A) tails (about 15–35 nucleotides) in healthy PBMCs, contrasted by smeared bands indicative of heterogeneous longer tails (about 35–105 nucleotides) in DLBCL cells (**Figure 1E**). Notably, eukaryotic mRNAs generally require a minimum Poly(A) tail length of ~30 nucleotides for stability (17). The suboptimal tail length observed in PBMCs provides a mechanistic explanation for the observed translational inefficiency despite elevated RGC32 mRNA levels in these cells.

### 3.3 RGC32 promoted cell proliferation in DLBCL *in vitro* and *in vivo*

**Figure 2:**
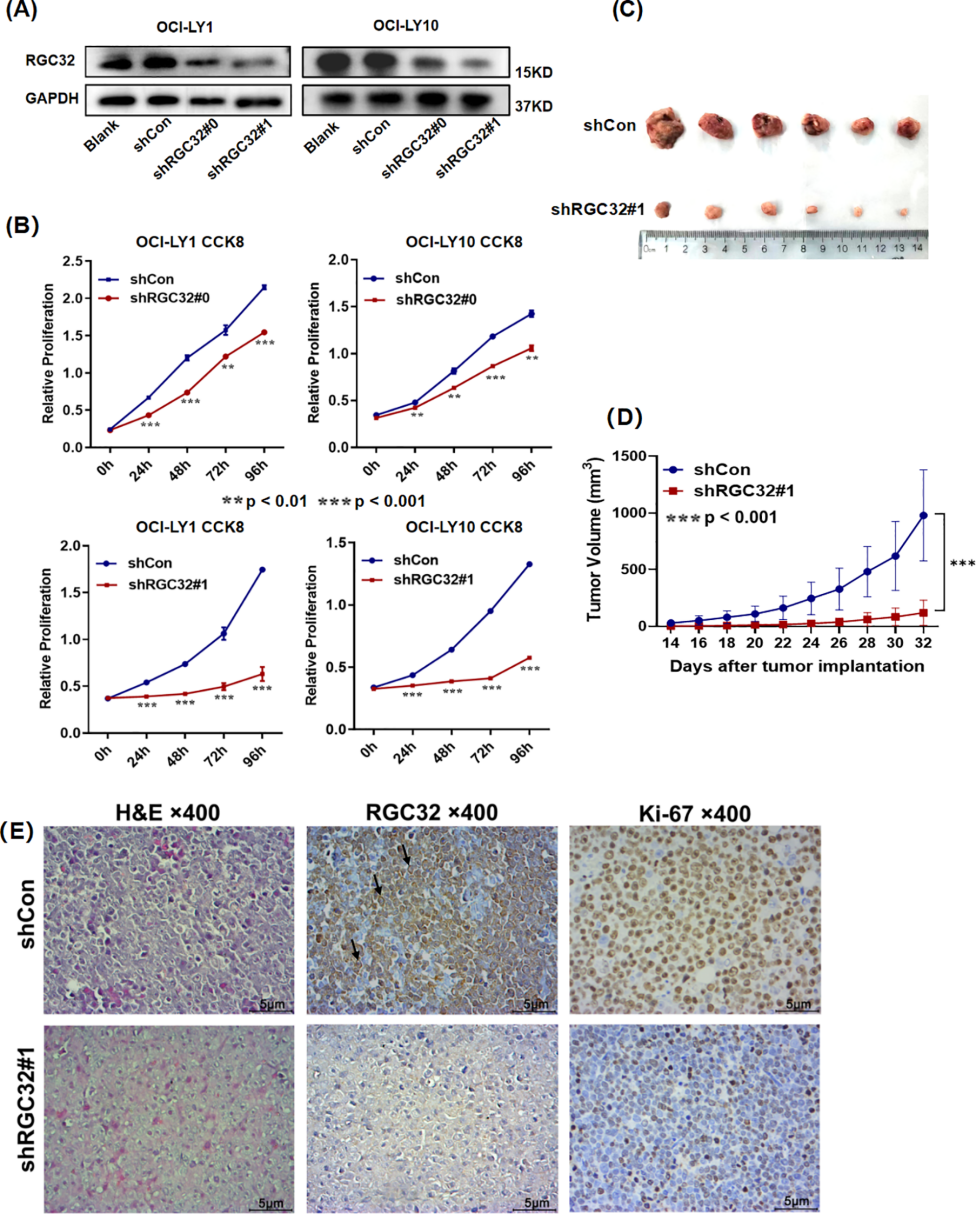
RGC32 promoted DLBCL growth *in vitro* and *in vivo*. **(A)** Western blot analysis was utilized to assess RGC32 protein expression in stably transduced OCI-LY1 and OCI-LY10 cell lines compared to vector control groups. **(B)** Silencing of RGC32 substantially inhibited proliferative capacity of both cellular models *in vitro*. **(C, D)** Xenograft models implanted with shRGC32-transfected cells exhibited significantly diminished tumor growth parameters relative to shControl cohorts, with consistent results observed in both tumor volume measurements (n = 6 per group). **(E)** Histopathological evaluation through H&E and IHC analysis demonstrated differential expression patterns of RGC32 and the proliferation marker Ki-67 in tumor xenograft specimens. Bar = 5μm.

To further investigate the biological function of RGC32 in DLBCL, we initially transfected three lentiviruses expressing shRNA into OCI-LY1 and OCI-LY10 cell lines to inhibit RGC32 expression. The knockdown efficiency was determined by western blot and data showed that shRGC32#0 and #1 reduced RGC32 protein level successfully in contrast to shControl (**Figure 2A**), of which shRGC32#1 exhibited better efficacy. *In vitro* experiments revealed that DLBCL cells with stable shRGC32 transfection showed significant growth retardation compared with their control counterparts. Particularly, lentiviral construct shRGC32#1 achieved superior growth suppression compared to shRGC32#0 across both OCI-LY1 and OCI-LY10 cellular models (**Figure 2B**). To further investigate the oncogenic potential of RGC32 *in vivo*, we established a xenograft tumor model by subcutaneously injecting SCID beige mice with OCI-LY1 cells carrying either shRGC32 or shControl constructs (six animals per experimental group). Tumor volume of mice implanted with shControl cells significantly grown faster and was larger in the end in comparison to mice with shRGC32 cells (**Figures 2C, D**). RGC32 and Ki-67 expressions were detected by IHC staining of the xenograft tumor. RGC32 and Ki-67 were detected higher expression in shControl tumor tissues than shRGC32 (**Figure 2E**). These results validated that RGC32 enhances tumor proliferation in DLBCL both *in vitro* and *in vivo*.

### 3.4 RNA-seq analysis revealed molecular mechanisms underlying the inhibition of proliferation after RGC32 knockdown

To examine the impact of RGC32 knockdown on the pathological effects of DLBCL, RNA-Seq analysis was conducted on OCI-LY1 cells stably transfected with either shRGC32 or shControl. The differential expression analysis comparing shRGC32 to shControl revealed 2,564 significantly differentially expressed genes (DEGs), with 1,104 being up-regulated and 1,460 down-regulated.

**Figure 3:**
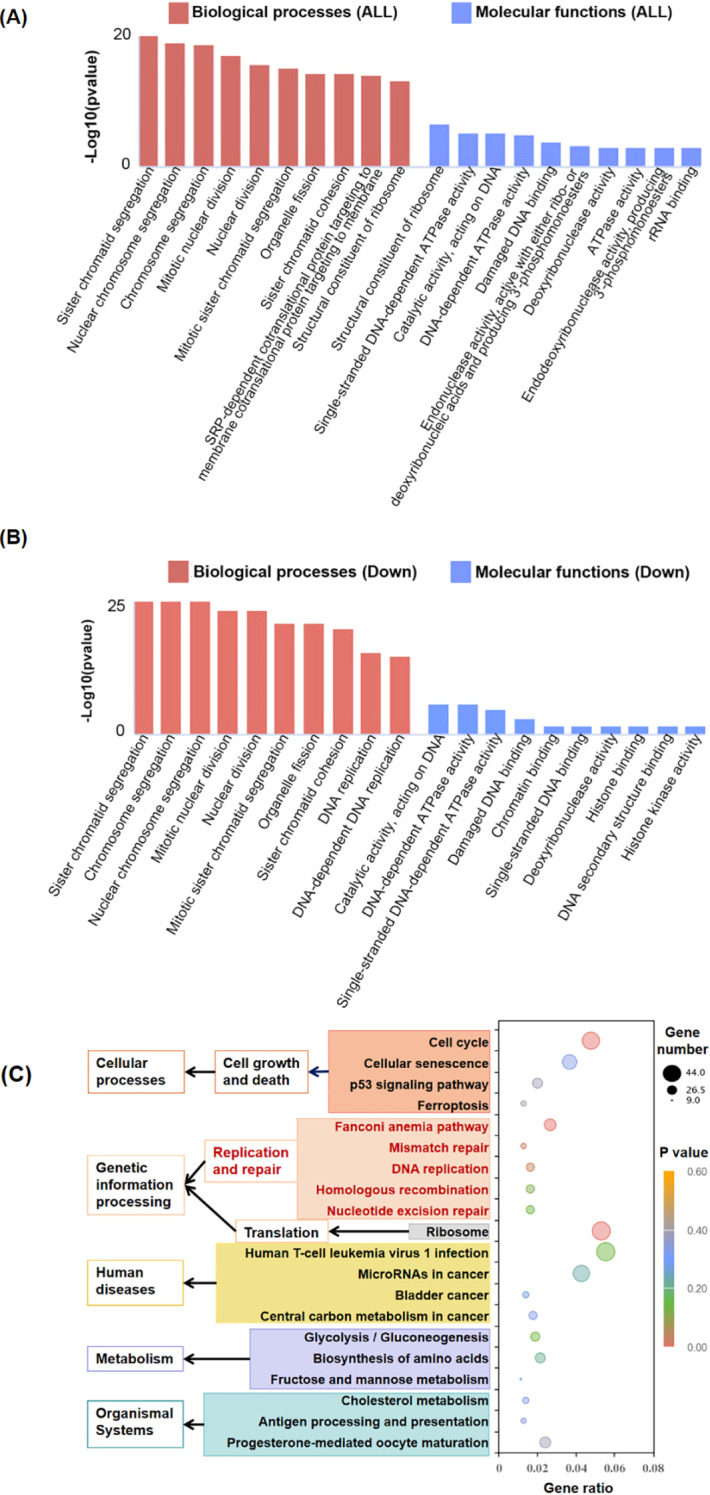
Comparison of gene expression in shRGC32 and shControl cell lines. **(A)** Top 10 enriched GO terms of BP and MF by all significantly regulated genes in shRGC32 compared to shControl cells, which sorted according to padj value. **(B)** Top 10 enriched GO terms of BP and MF by significantly down-regulated genes in shRGC32 compared to shControl cells, which sorted according to padj value. **(C)** The top 20 significantly enriched KEGG pathways clustered into different subcategories.

To investigate the functional characteristics of DEGs, we performed functional enrichment analysis through GO categorization. The ClusterProfile tool was utilized to analyze the regulated genes, revealing altered biological processes (BP) and molecular functions (MF) among the 2,564 DEGs. The ten most significantly enriched terms based on adjusted p-values are presented in **Figure 3A**. RGC32 showed a strong association with cell mitosis and DNA replication, particularly within the enriched BP terms of down-regulated DEGs (**Figure 3B**). Additionally, to elucidate the systemic biological implications of the experimental findings, pathway enrichment analysis through KEGG database was conducted. The investigation revealed 29 statistically significant pathways (p < 0.05), with the top 20 functionally distinct pathways being systematically classified (**Figure 3C**). Notably, molecular pathways governing cell growth and death as well as DNA replication and repair exhibited the most pronounced alterations following RGC32 knockdown. Both GO and KEGG analyses supported the observed phenotype of proliferation inhibition in cells with RGC32 knockdown.

### 3.5 Suppression of RGC32 expression arrested cell cycle progression at the G0/G1 phase in DLBCL cell lines

**Figure 4:**
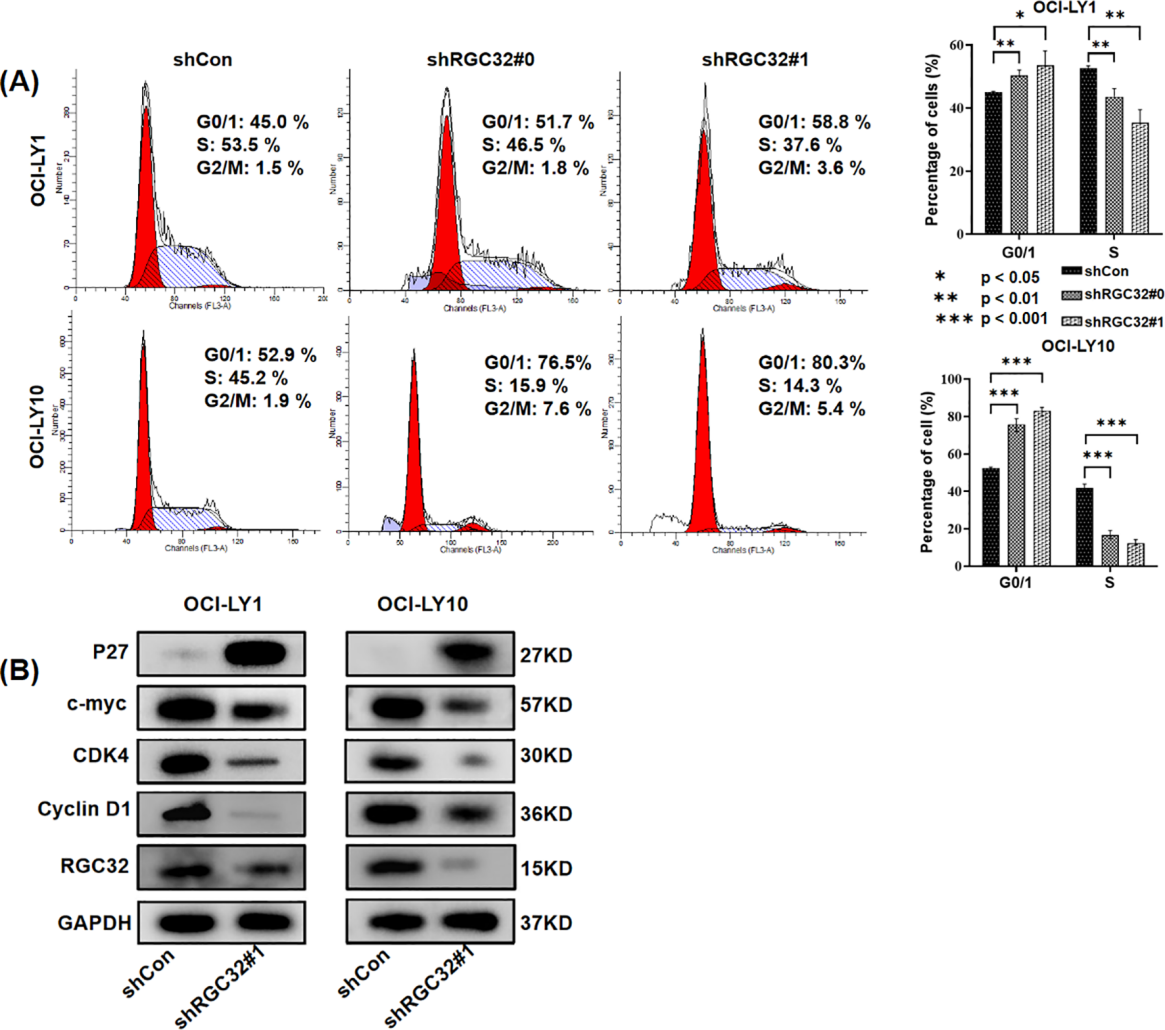
RGC32 knockdown resulted in cell cycle arrest in DLBCL cells. **(A)** RGC32 knockdown induced cell cycle arrest at G0/1 phase in OCI-LY1 and OCI-LY10 cells. **(B)** RGC32 knockdown regulated the expression of proteins associated S-phase entry from G1 phase.

As mentioned above, RNA-seq confirmed that RGC32 facilitated cell mitosis and the progression of the cell cycle, aligning with earlier studies (3). But the regulation mechanism of RGC32 on the cell cycle is controversial. Depletion of RGC32 has been shown to mediate G0/G1 phase cell cycle arrest across multiple cellular models, such as in aortic smooth muscle cells (4) and human pulmonary carcinoma (18). Otherwise, RGC32 could also disrupt the G2/M checkpoint such as in glioma cells (19) and renal tubular epithelial cell (20). In DLBCL cell lines, we found both OCI-LY1 and OCI-LY10 cells with RGC32 hypo-expression were halted in the G0/G1 phase, accompanied by a reduction in the S phase of the cell cycle (**Figure 4A**). In addition, western-blot explored the expression of proteins associated S-phase entry from G1 phase. Regulatory component of the cyclin D1-cyclin dependent kinase 4 (CDK4) complex (21) required for G1/S transition and c-myc (22), a nuclear phosphoprotein which could promote cell cycle progression, were all reduced by RGC32 knockdown (**Figure 4B**). Respectively, P27 (23) was up regulated after RGC32 knockdown (**Figure 4B**), which was a CDK4 inhibitor involved in G1 phase arrest.

### 3.6 RGC32 promotes DNA damage repair by ATM/ATR/CHK1 in DLBCL

**Figure 5:**
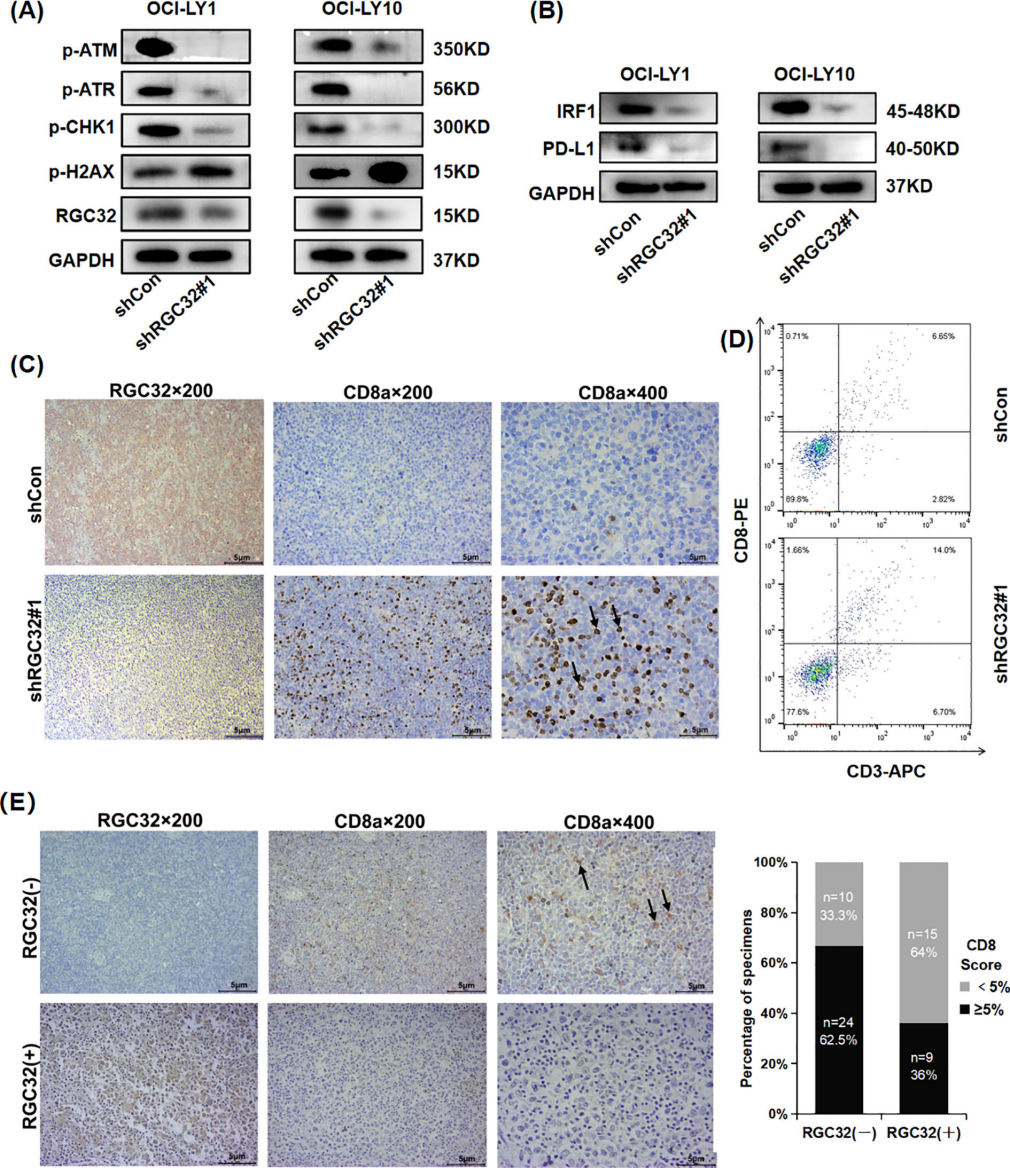
RGC32 knockdown promoted DNA damage and CD8+ T cells. infiltration in DLBCL TME. **(A, B)** Western blot was conducted to assess the protein levels of phosphorylated (p) ATM/ATR/CHK1, p-H2AX, IRF1 and PD-L1 in cells transfected with shControl and shRGC32. **(C, D)** *In vivo*, IHC and flow cytometry showed knockdown of RGC32 increased CD8 expression. **(E)** IHC images represented the association between expressions of CD8 and RGC32 in DLBCL tissues. Bar = 5μm.

In addition to its extensively reported role in cell cycle modulation, the RNA-seq profiling revealed that suppression of RGC32 expression significantly attenuated several DNA damage response mechanisms, encompassing Fanconi anemia pathway, mismatch repair (MMR), DNA replication, homologous recombination (HRR) and nucleotide excision repair (NER) (**Figure 3C**). These coordinated alterations suggest RGC32’s potential involvement in orchestrating common regulatory nodes within the DNA damage response network, thereby enabling simultaneous modulation of multiple repair cascades. This regulatory capacity may involve interaction with ataxia telangiectasia mutated (ATM) and ataxia telangiectasia and Rad3 related (ATR) - two key serine/threonine protein kinases serving as primary sensors in genomic surveillance systems. Phosphorylation of ATM and ATR followed by Checkpoint Kinase 1 (CHK1), a cell-cycle checkpoint kinase, engaged in the suppression of DNA replication and mitosis, while additionally facilitating DNA repair and recombination (24). The RNA-seq results also showed the expression levels of the ATM, ATR and CHK1 genes were all reduced following RGC32 knockdown (Additional file 3). Therefore, to investigate whether the ATM/ATR/CHK1 pathway was participating in DDR regulation caused by RGC32, phosphorylation of ATM, ATR and CHK1 were examined by western-blot and all of them were obviously suppressed by RGC32 knockdown (**Figure 5A**). Furthermore, inhibition of DDR can cause the cumulation of DNA damages (24). P-H2AX is a sensitive index for DNA damage, especially for double-strand breaks (DSBs) which is the most critical type of genotoxic stress for antigen-presenting (25). In this study, p-H2AX was significantly up-regulated following RGC32 knockdown (**Figure 5A**), which partly reflected the increase in DNA damage accumulation. In summary, RGC32 could regulate the ATM/ATR/CHK1 pathway and decrease the DNA damage load.

### 3.7 RGC32 inhibited CD8+ T cell infiltration in DLBCL TME

Previous research has described DNA damage and genomic instability by DDR defects or DDR inhibitors in tumor cells can lead enhancing the level of the TMB, which is an important source of neoantigens for tumors (9). Neoantigens are presented by major histocompatibility complex (MHC)-I on tumor cells and subsequently enhance the antigen presentation process to increase CD8+T cells in TME (10). PD-L1 is another key molecule in the tumor immunity, which binds to PD-1 on the surface of CD8+ T cells, inhibiting the proliferation, activation and cytokine secretion of CD8+ T cells (25). In DDR, PD-L1 activation requires ATM/ATR/CHK1 activity via Interferon regulatory factor 1 (IRF1) pathway (9, 26). The RNA-seq results also showed the expression levels of the IRF1 and PD-L1 genes were reduced after RGC32 knockdown (Additional file 3). Furthermore, Western-blot confirmed that the knockdown of RGC32 resulted in decreased protein levels of IRF1 and PD-L1 (**Figure 5B**), which was consistent with ATM/ATR/CHK1 inhibition. Moreover, KEGG enrichment results showed that RGC32 was related to antigen processing and presentation (**Figure 3C**), so we speculated that RGC32 could affect the level of CD8+T cells in TME of DLBCL.

**Figure 6:**
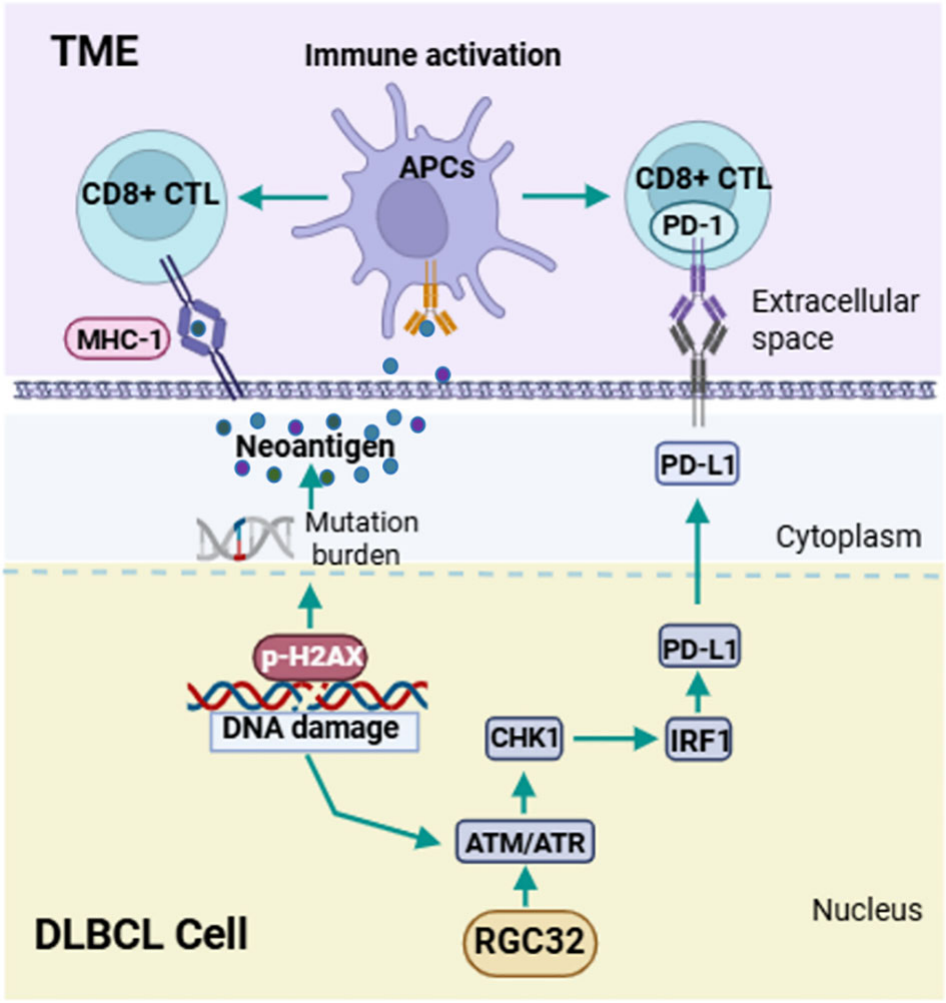
Schematic diagram of pathways regulated by RGC32 in DLBCL. RGC32 promotes DNA damage repair in DLBCL cells by activating the ATM/ATR/CHK1 pathway. The reduction of DNA damage leads to a decrease in tumor neoantigen load, and the ATM/ATR/CHK1 pathway can activate IRF1 to promote PD-L1 expression. Both of these aspects could inhibit the infiltration of CD8+ T lymphocytes in the tumor microenvironment, thereby promoting the occurrence and development of DLBCL.

CD8+ T cells are the primary mediator in the anticancer immunity of TME, which can be enhanced by the accumulation of DNA damages (27). We asked if DDR defects by RGC32 knockdown were also associated with a CD8+ T cell immune response. The presence of intratumoral CD8+ T lymphocytes were assessed by IHC and flow cytometry in previously described xenograft samples of shRGC32 or shControl OCI-LY1 cells. A high expression of intratumoral CD8+ T lymphocytes with shRGC32 xenograft tumors was identified (**Figures 5C, D**). To determine whether CD8+ T cell expression was also related with RGC32 in DLBCL tumors, we performed IHC analysis of the cohort of 58 DLBCL patients’ tissues scored for high or low RGC32 expression groups in front part of this article. A previously published cut-off of 5% were used to define CD8 positive (28). A statistically significant negative association between the CD8 expression and the positive score of RGC32 was observed (p = 0.023, **Figure 5E**). Therefore, we concluded that RGC32 influence DNA damage repair and modified the infiltration of CD8+ T cells in DLBCL TME (**Figure 6**).

## 4 Discussion

In the current study, we have, for the first time, clarified the increased expression and oncogenic function of RGC32 in DLBCL. RGC32 was found to be up-regulated in DLBCL and showed a correlation with patient prognosis. Inhibiting of RGC32 demonstrated potential therapeutic benefits in DLBCL by reducing cell proliferation, hindering cell cycle progression, impairing DNA damage repair, and increasing CD8+ T lymphocyte infiltration. These promising results hold significant implications for developing new treatment strategies aimed at enhancing long-term survival for patients with DLBCL.

Abnormal expressions of RGC32 have been found in some solid tumors, such as high expression in colorectal (6, 29), pancreatic (5, 30), breast (31), Renal Cell Carcinoma (32), ovarian (33), but low expression in astrocytoma (19, 34) and adrenocortical carcinoma (35). In our study, consistent with most studies in tumors, RGC32 expression was also found to be increased in DLBCL, and elevated levels of RGC32 were linked to a poor prognosis. But, there was a notable discrepancy between the mRNA and protein expression levels in DLBCL cell lines. This difference is likely to be related to the length of the Poly(A) tail. Poly(A) tails exert a significant role in the regulation of eukaryotic gene expression, and the majority of their functions are dependent on their binding to Poly(A)-binding proteins (PABPC) (16). A suitable Poly(A) length is necessary for efficient transcription. First of all, this tail should long enough to accommodate a single PABPC, who’s full-length is about 30 nucleotides. If the Poly(A) length is less than 30 nucleotides, the mRNA will undergo active degradation (16). On the other hand, instead of long tails, short Poly(A) tails are associated with longer half-lives and highly expressed mRNAs (16). Experimental evidence from Xenopus laevis oocyte injection studies demonstrates this length-dependent functionality: poly(A) tails exceeding 32 nt achieve comparable translational efficiency to native globin mRNA containing 149-nt poly(A) tails (36). The length of ploy Poly(A) tails is not continuous, but lengthens in increments of 30 nt, which is consistent with the full-length of PABPC binding to the poly(A) tail (37). In our study, RGC32 mRNA PAL in PBMCs was too short to maintain the stability and transcriptional activity, however, RGC32 mRNA PAL in DLBCL cells had a reasonable length of tails, about 1–3 PABPCs, for the effective transcription. Until recently, the research on how the PAL of a single mRNA regulated remained limited, partly because analysis of Poly(A) tail length of specific mRNA was difficult to perform. For RGC32, one study discovered that the mutation of a solitary Pumilio binding element (PBE), adjacent to the polyadenylation signal, gave rise to an increase in the length of PAL (15).

The expression profile of RGC32 exhibits considerable heterogeneity across various tumor types, a phenomenon that may be attributed to distinct tumor-specific mechanisms underlying its regulation of cellular proliferation. In most tumors with RGC32 high expression, it performed as a cell cycle regulatory factor and promoted cell proliferation (5, 6, 30–33). RGC32 functions as a substrate and regulator of p34CDC2 activity and can cause cell cycle arrest of tumor cells at G0/G1 phase (4). But over-expression of RGC32 suppressed glioma cell growth, probably by forming a protein complex with polo-like kinase 1 (PLK1) and inducting cell cycle arrest at G2/M (19). Present study found that RGC32 inhibition suppressed cell proliferation, and promoting arrest of cell cycle at G0/G1 phase in DLBCL cells. Moreover, KEGG analysis revealed that cell cycle regulation might be the most important mechanism underlying the proliferation inhibition of RGC32 knockdown. The above results were consistent with previous findings (5, 6).

In addition to its definite role in regulating the cell cycle in tumors, our study first reported that RGC32 was closely associated with the DNA damage repair. Dysfunctions of the DNA damage repair result in the genomic instability and is recognized as a character of many solid tumors and leukemia (38). If the damage is excessive, cells no longer expend energy to repair the damage and may progress to apoptosis or senescence (8). At the same time, DNA damages have been shown to be a promising predictor of sensitive to DNA damage drugs and immune checkpoint inhibitors (ICIs) in solid tumors (39, 40). DNA damage checkpoints are DNA damage signals, which can be activated by DNA damage to regulate the cell cycle and promote DNA repair (8). ATM/ATR/CHK1 pathway is the most important DNA damage checkpoint signal (8). This pathway is activated or up-regulated in many cancers, inhibition of any molecule of this pathway can increase sensitivity to DNA damage drugs and promote tumor cell apoptosis (41). Our study found that RGC32 had significant positive effects on this ATM/ATR/CHK1 pathway, so we speculated that the inhibition of DLBCL cells proliferation caused by inhibition of RGC32 partly depended on the inhibition of this pathway.

As comprehension of the mechanisms involved in cancer therapy grows, numerous studies indicate that DNA damage response influences both the TME and the effectiveness of ICIs (9, 42). CD8+ T cells are the primary mediators of adaptive anticancer immunity (43) and loss of DDR functions can affect CD8+ T cells recruitment in several ways (9). DDR dysfunctions result in intracellular DNA fragment accumulation and enlargement of somatic mutations, which also increase neoantigen accumulation (44). Neoantigens could increase the antigen presentation process of dendritic cells (DCs), which is necessary for priming effective CD8+ T cells recruitment and more TILs are recruited at the same time (9). Next neoantigens must be directly presented by MHC-I for recognition and killing by primed CD8+ T cells (9). What’s more, DNA damage can enhance the expression of PD-L1, facilitating tumor evasion and hindering the recruitment of CD8 T cells, partly by activating ATM/ATR/CHK1 pathway through IRF1 signaling (11). Until recently, many studies have identified that inhibitors of components in the ATM/ATR/CHK1 pathway in cancers can down-regulate PD-L1 expression and promote the immune microenvironment (26, 45, 46). In our study, RGC32 down-regulation resulted in DDR dysfunctions and increased DNA damage accumulations. At the same time, PD-L1 expression was also inhibited along with down-regulation of the ATM/ATR/CHK1 pathway and IRF1. Therefore, subsequent investigations demonstrated that RGC32 downregulation enhances CD8+ T cell recruitment, as evidenced by our experimental findings. RGC32 regulating immunity has also been reported in other tissues, but mechanisms are different. Colon cancer cells promoted RGC32 expression in macrophages, which subsequently enhanced macrophage migration and promoted tumor progression through paracrine mechanisms (29). CD4+ and CD8+ T cells purified from the spleen of RGC32 knockout mouse exhibit greater proliferation than those from wild-type mice (47). Therefore, RGC32 exerts a synergistic effect in inhibiting tumor immunity, not only by suppressing the antigen-presenting capacity of CD8+ T lymphocytes in tumor cells, but also by hampering the proliferation of immune cells within the tumor microenvironment. However, whether the two mechanisms act simultaneously has not been studied and this is the work that we will do in the future.

## 5 Conclusion

Our research demonstrated the elevated expression and oncogenic function of RGC32 in DLBCL. RGC32 was highly expressed in DLBCL and associated with adverse patient prognosis. RGC32 promotes tumor progression in DLBCL by enhancing DDR signaling pathways and inhibiting the recruitment of CD8+T lymphocytes within the tumor microenvironment. Taking together, our findings raise the likelihood that RGC32 emerges as a promising regulator of progression and a potential therapeutic target in DLBCL.

## Data Availability

The data presented in the study are deposited in the NCBI GEO repository, with accession number GSE301951.
