# Supplementary material for: Response gene to complement 32 promotes tumorigenesis by mediating DNA damage repair and inhibits CD8+ T cells infiltration in diffuse large B-cell lymphoma
Source: Front Immunol. 2025 Jul 16;16:1591615. doi: 10.3389/fimmu.2025.1591615 (PMC12309393; doi:10.3389/fimmu.2025.1591615)
Supplement: Supplementary file 2 [file DataSheet2.pdf]

Additional file 2
Supplemental Methods
Clinical specimens
This study analyzed 112 biological specimens, including 80 paraffin-embedded tissue samples from diffuse large B-cell lymphoma (DLBCL) patients and 32 control samples with reactive lymphoid hyperplasia (RHL). The 80 tumor specimens were derived from a cohort of newly diagnosed DLBCL patients, selected based on clinical staging and treatment-naive status to minimize confounding variables. The 32 control specimens included non-tumor lymphoid tissues (e.g., reactive lymph nodes) matched for age, sex, and anatomical site to account for microenvironmental and technical variability. The sample sizes were determined using power analysis ($\alpha=0.05$, $\beta=0.2$) based on preliminary data showing significant differences in RGC32 expression between tumor and controls (effect size=1.2). The DLBCL cohort consisted of 45 female and 35 male patients (median age: 58 years; range: 25-85 years) whose diagnoses were pathologically confirmed according to WHO classification criteria for lymphoid malignancies1. Specimens were collected over a decade (2011-2022) from the hospital archives.

For comparative analysis, peripheral blood mononuclear cells (PBMCs) were isolated from healthy donors using standardized Ficoll-Hypaque density gradient centrifugation. Healthy donor PBMCs (n=8) served as a baseline for immune cell profiling, as they lack tumor-induced alterations. Therefore, Healthy donor PBMCs was used for comparison with lymphoma cell lines. The experimental protocol received ethical approval from the Institutional Review Board of Shandong Provincial Hospital Affiliated to Shandong University (SPHASU), with all procedures complying with Declaration of Helsinki principles.

Immunohistochemistry (IHC) and hematoxylin-eosin (H&E) staining
This study employed standardized immunohistochemical protocols for paraffin-embedded human and murine tissues. Following deparaffinization and rehydration of 4-μm sections, antigen retrieval was performed using pressurized 1x EDTA buffer with subsequent 2-hour cooling. Endogenous peroxidase activity was quenched with 3% H_2O_2 (15 min), followed by non-specific blocking with 5% BSA (30 min). Primary antibody incubation included anti-RGC32 (orb2372, Biorbyt, 1:200) and species-specific CD8a antibodies (human: A0663, ABclonal 1:200; murine: GB114196, Servicebio 1:500) at 4°C overnight, supplemented with anti-Ki67 (GB121141, Servicebio 1:300) for murine specimens. Post-rinsing, sequential incubations with biotinylated secondary antibody (37°C, 30 min) and streptavidin-HRP complex (SABC, 30 min) preceded DAB chromogenic development (1-3 min) and hematoxylin counterstaining. Digital imaging using an Olympus BX63 microscope captured five representative fields at ×200/400 magnifications. Blinded dual-observer analysis employed distinct evaluation metrics: RGC32 expression was quantified by multiplying proportion scores (0-4 scale: 0% = 0; 1-25% = 1; 26-50% = 2; 51-75% = 3; 76-100% = 4) with intensity grades (0-3: negative = 0, weak = 1, moderate = 2, strong = 3), while CD8a+ infiltration adopted the ≥5% positivity threshold established by Rajnai et al[1]. Murine tumor validation included parallel H&E staining and 4% PFA-fixed paraffin processing, ensuring methodological consistency across experimental models.

Western blotting
Western blot analysis was performed using optimized protocols. DLBCL cells were harvested, washed three times with PBS, and lysed in ice-cold RIPA buffer (Shenergy Biocolor, China) supplemented with 1× phosphatase inhibitor cocktail (PhosSTOP; Roche, Germany). Protein quantification was performed using BCA assay before

loading 30 μg aliquots of total protein onto 10% Tris-Glycine gels (TGXTM FastcastTM, Bio-Rad, USA) for electrophoretic separation. Following transfer to PVDF membranes (0.45 μm, Millipore, USA) using semi-dry transfer system, membranes underwent sequential blocking steps: initial 2h blocking with 5% BSA at room temperature followed by overnight primary antibody incubation at 4°C. Antibody specifications included: RGC32 (1:1000, NBP2-93098, NOVUS, USA) and GAPDH (1:2000, Zhongshan Goldenbridge, TA-08, Beijing, China). Cell Signaling Technology antibodies: c-myc (18583), Cyclin D1 (2922), CDK4 (12790), p27 (3688), p-ATM (5883), p-ATR (2853), p-CHK1 (2348), IRF1 (8478), PD-L1 (13684), p-H2AX (9718) (all 1:1000). Membranes were subsequently probed with HRP-conjugated secondary antibodies (1:5000, Zhongshan Goldenbridge, ZB-2306, China) for 1h at room temperature after TBST washing. Chemiluminescent detection was achieved using ECL reagent (Millipore, USA) with image acquisition on FluorChem E system (Protein Simple, USA). GAPDH served as loading control for triplicate experiments.

Poly(A) Tail (PAT) length assay

This assay was carried out by Poly(A) Tail-Length Assay Kit (76455, Affymetrix, USA) . Total 0.5 μg RNA samples were incubated with tail enzyme mix at 37°C for 60 minutes to add poly(G/I) tails. Next to reverse transcribe the poly(G/I) tailed RNA, the production mixed with RT Enzyme Incubate at 44°C for 60 minutes, 92°C for 10 minutes and at 4°C hold. Diluted RT sample mixed with RGC32 and Actin Gene-Specific PCR Forward Primers, Universal PCR Reverse Primers and HotStart-IT® Taq DNA Polymerase for PCR amplification using two-step PCR (94°C for 2 minutes, 30-35 cycles of: 94°C for 10 seconds 60°C for 30-60 seconds, 72°C for 5 minutes, 4°C hold). RGC32 Gene-Specific PCR Forward and Reverse Primers were used as control. The size of PCR products was assessed by 2% agarose TAE gel and visualized by a chemiluminescence imaging system (azure biosystems C300). RGC32-specific primers used were as follows: forward, 5′-GCTTTTATGAAAATATTTGTAATTAATTATATATAGTTGG; reverse, 5′-TCAGTTCAAATTTTATTTGTATAC. The method of Poly(A) tail-length determination was shown in Fig.1

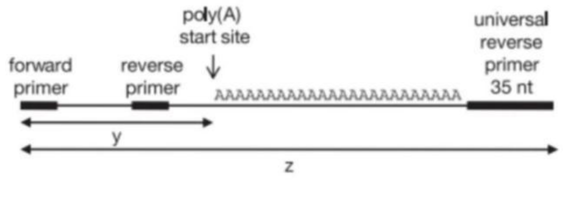

Fig.1 Poly(A) tail-length determination. A-tail length is (z - y - 35) where z can vary based on gel results (y = 100).

Cell transfection

Lentivirus vectors either encoding sh-RGC32 or control were from Genechem (Shanghai, China). The following RNAi sequences were used: sh-RGC 0# GTCACTCCTCAGAAAGCTAAA; sh-RGC 1# CGGAGAGTGCAGATTCACTTT, sh-RGC 2# GAACAGACGATCCATGCTAAT. The lentiviral transduction procedure was performed following the standardized protocol provided by the manufacturer. To establish stable cell lines, transfected populations underwent antibiotic selection with 2 μg/mL puromycin (Genechem, China) for 7-14 days. Transduction success was quantitatively verified through Western blot analysis of target protein expression levels.

Quantitative real-time PCR

Total RNA isolation was performed with RNAiso Plus reagent (TaKaRa, China). First-strand cDNA synthesis was performed with reverse transcriptase kits (Vazyme, China) according to manufacturer's protocols. Quantitative PCR amplification was executed on a Light Cycler 480II system (Roche, Switzerland) employing SYBR Green chemistry (Vazyme, China). The primer pair targeting RGC32 consisted of: forward 5′-CGACTCGGAGAGTGCACATTC and reverse 5′-TTTCTGAGGAGTGACAGTGGC. Gene expression levels were quantified using the $2^{-\Delta\Delta CT}$ method with triplicate technical replicates for each experimental sample. All reactions were performed in triplicate to ensure technical reproducibility.

Cell proliferation assay and cell cycle assay

The proliferative capacity of DLBCL cells was assessed through CCK-8 methodology (Yeasen, China). Experimental cell populations subjected to specific interventions were cultured in 96-well microplates for 24-96 hours prior to analysis. Cellular metabolic activity was quantified by introducing 10μl CCK-8 reagent into each well, followed by 4-hour incubation at 37°C. Optical density measurements at 450 nm wavelength were subsequently obtained using a Thermo Scientific Multiskan GO Microplate Spectrophotometer.

The cell cycle distribution was assessed through flow cytometry. For this analysis, DLBCL cells were harvested and washed with phosphate-buffered saline (PBS) prior to overnight fixation at -20°C using 70% ethanol. Cell cycle profiling was subsequently conducted employing a Navios FACS-240 flow cytometer (Beckman Coulter, USA), with data acquisition and analysis performed according to standardized protocols for cell cycle phase determination.

***In vivo* xenograft study**

Animal experimental protocols in this study were conducted in compliance with SPHASU Animal Care and Use Ethics Committee standards and ARRIE guidelines. Female SCID mice (3-week-old) acquired from a certified laboratory animal center were acclimatized for seven days in controlled pathogen-free conditions prior to experimentation. Subjects were randomly allocated into two experimental cohorts receiving subcutaneous injections (100μl suspension) in the right hind limb containing 1×10^7 DLBCL cells - either transfected with control vectors or RGC32-knockdown constructs. Tumor growth metrics were collected bi-daily using digital calipers, with volumetric calculations employing the ellipsoid approximation formula: $V = (a \times b^2) \times 0.5236$ (a = long axis, b = short axis). All measurements were recorded by researchers blinded to treatment group assignments. Post-sacrifice analyses included histopathological evaluation and flow cytometric characterization of excised tumor masses.

Tumor-infiltrating lymphocytes analysis

The shRGC32 and shCon established xenograft tumors were cut into small enough pieces, and add 10 ml of digestion medium (collagenase and HEPES) (Solarbio, China) to the centrifuge tube placed at 37℃for 1 hour. Twenty ml of FACs Buffer was added to the centrifuge tube, immediately after the digestion. Subsequently, the digested tissue fluid was filtered through the funnel and discard the tissue precipitation. Then the digested tissue fluid was centrifuged at 2000 rpm for 10 min. The precipitate was suspended in 4 ml of 40% percoll (Solarbio, China) and slowly added to a new 15 ml centrifuge tube with 4 ml of 80% percoll, centrifuged at 2500 rpm, up 6 down 2, for 30 min. The inter layer cells were carefully drawn to a new centrifuge tube and washed with 8 ml FACs Buffer. Mononuclear cell samples were performed for cell counting and ensure that the number of cells per tube is in the range of 105 -106. Add 0.3μl CD8a Flow antibody (PE-65069, Proteintech) or 0.5μl CD3 Flow antibody (APC-65077, Proteintech) to each tube and stained at 4℃ light avoidance for 30 min. Finally, it was

tested by FACS- 240 Navios Flow Cytometer (Beckman Coulter Inc. USA) after centrifuged and suspended by 600μl PBS.

References

1. Quintanilla-Martinez L. The 2016 updated WHO classification of lymphoid neoplasias. Hematol Oncol. 2017;35 Suppl 1:37-45. doi:10.1002/hon.2399

2. Rajnai H, Heyning FH, Koens L, Sebestyén A, Andrikovics H, Hogendoorn PC, et al. The density of CD8+ T-cell infiltration and expression of BCL2 predicts outcome of primary diffuse large B-cell lymphoma of bone. Virchows Arch. 2014;464(2):229-239. doi:10.1007/s00428-013-1519-9
