# Supplementary material for: Response gene to complement 32 promotes tumorigenesis by mediating DNA damage repair and inhibits CD8+ T cells infiltration in diffuse large B-cell lymphoma
Source: Front Immunol. 2025 Jul 16;16:1591615. doi: 10.3389/fimmu.2025.1591615 (PMC12309393; doi:10.3389/fimmu.2025.1591615)
Supplement: Supplementary file 3 [file DataSheet3.pdf]

Additional file 3

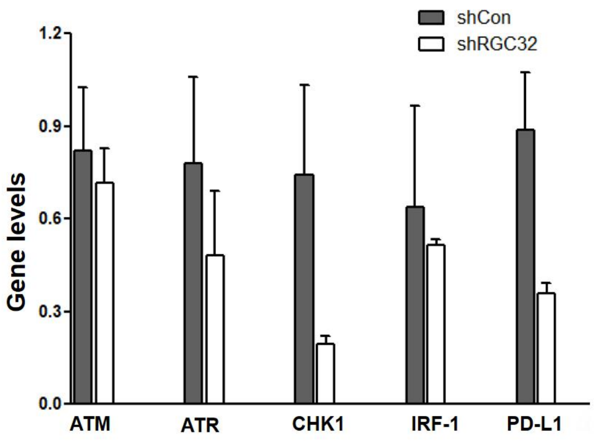

Figure S1 The RNA-seq results demonstrated that the expression levels of the ATM, ATR, CHK1, IRF1 and PD-L1 genes were all reduced following RGC32 knockdown.
